# Academic General and Subspecialty Pediatric Promotion Timelines and Lifetime Earnings

**DOI:** 10.1001/jamanetworkopen.2025.40875

**Published:** 2025-10-31

**Authors:** Lubaina Ehsan, Murad Almasri, Krittika Joshi, Bilal Celik, Ye Hong, Joshua Daily

**Affiliations:** 1 Division of Cardiology, Department of Pediatrics, University of Arkansas for Medical Sciences/Arkansas Children’s Hospital, Little Rock; 2 Department of Economics, Sam M. Walton College of Business, University of Arkansas, Fayetteville

## Abstract

**Importance:**

Differences in academic promotion timelines may contribute to long-standing disparities in pediatric physician compensation and impact workforce sustainability.

**Objective:**

To evaluate the association between promotion timing and the net present value (NPV) of lifetime earnings for academic general pediatricians and pediatric subspecialists across salary percentiles and career lengths.

**Design, Setting, and Participants:**

This cross-sectional study using a financial modeling strategy examined 2024-2025 compensation data from the Association of Administrators in Academic Pediatrics. The NPV analysis was conducted for 29 pediatric fields across 4 promotion scenarios (early promotion, baseline promotion, stalled promotion, and no promotion), 3 salary percentiles (25th, 50th, and 75th), and 3 career lengths (standard, part time, and early retirement).

**Exposure:**

Promotion timeline and pediatric subspecialty.

**Main Outcome and Measures:**

The outcome was NPV of lifetime earnings as measured in constant 2025 dollars.

**Results:**

A total of 19 111 academic pediatricians and pediatric subspecialists were included from the compensation dataset. Early promotion consistently yielded the highest NPV across all subspecialties and salary percentiles (median NPV, $7 621 223.50), while remaining at the assistant professor rank for an entire career was associated with the lowest NPV (median, $6 967 428.72). Baseline promotion NPV peaked at a mean (SD) of $9 364 986.25 ($695 347.50), while no promotion NPV was significantly lower (mean [SD], $7 093 248.02 [$475 237.71]). Stalled promotion reduced the median NPV by $500 000 to $1.5 million; no promotion led to losses exceeding $2 million to $3 million in high-paying subspecialties, such as cardiology, critical care, and neonatology. Sensitivity analyses and Monte Carlo simulations found that delayed promotion was negatively associated with lifetime earnings under all tested scenarios.

**Conclusions and Relevance:**

In this cross-sectional study of academic pediatricians, promotion timing was associated with lifetime earnings and often more influential than subspecialty or salary percentile. The findings suggest that physicians who were never promoted may need to work an additional 10 years to match the earnings of peers promoted via a baseline promotion trajectory. The findings highlight the financial consequences of delayed advancement and underscore the need for institutional efforts to support timely and equitable promotion across the pediatric workforce.

## Key Points

**Question:**

What is the association between academic promotion timelines and lifetime earnings of general pediatricians and pediatric subspecialists?

**Findings:**

In this cross-sectional study of 19 111 academic pediatricians, earlier promotion was consistently associated with significantly higher net present value of lifetime earnings, regardless of subspecialty or salary percentile. Physicians who were never promoted faced financial losses exceeding $2 million to $3 million in some fields and would need to work approximately 10 additional years to match the net present value of peers on a baseline promotion trajectory.

**Meaning:**

These findings suggest that promotion timing is a critical determinant of long-term financial outcomes in academic pediatrics and underscore the need to address disparities in advancement to strengthen and sustain the pediatric workforce.

## Teaser

This cross-sectional study evaluates the association between promotion timing and the net present value of lifetime earning for academic general and subspecialty pediatricians across their careers.

## Introduction

Academic promotion plays a pivotal role in shaping careers, serving as both a marker of achievement and a determinant of salary benchmarks, leadership opportunities, and long-term financial outcomes.^1,2,3,4,5^ It is a critical milestone not only for career advancement and leadership opportunities but also for its substantial impact on lifetime earnings.^1,6^ Despite its importance, limited empirical data exist on how the timing of promotion influences the net present value (NPV) of lifetime earnings in academic pediatrics.^7,8,9,10^

Prior research has shown that pediatric subspecialties emphasizing inpatient and procedure-based care, such as neonatology, critical care, cardiology, and gastroenterology, tend to offer higher lifetime earning potential than other pediatric fields.^11,12,13^ Although academic promotion is associated with increased prestige, leadership opportunities, and salary advancement, its effect on the NPV of lifetime earnings has only recently been explored in pediatrics. For example, Catenaccio et al^14^ modeled the association of gender-based differences with promotion timing, showing that delayed advancement could significantly reduce long-term earnings. Their analysis focused on 1-year delays and early-career stagnation in promotion to associate professor. Our study builds upon this work by modeling full-career trajectories, including early promotion, baseline promotion, stalled promotion, and no promotion, across all academic pediatric subspecialties and compensation percentiles.

Net present value analysis is a widely accepted and rigorous financial method for calculating and comparing the lifetime earnings of academic physicians based on the principle that a dollar today holds more value than in the future.^11,15,16,17,18^ This concept is particularly pertinent when evaluating physician careers, which often span several decades.^11,13,16,17,18^ Recent applications of NPV in academic medicine have informed key career decisions. A national analysis comparing MD and MD-PhD training pathways found that MD-PhD graduates had lower lifetime earnings at approximately 7% less than MDs.^19^ Within pediatrics, NPV analysis has shown a wide variation in subspecialty earnings.^11,17^ Net present value comparisons between pediatric and adult subspecialties have shown that pediatric subspecialists earn less over their careers, largely due to lower initial salaries, slower wage growth, and a higher reliance on Medicaid reimbursement.^4,20,21^ In pediatric medicine, in which most subspecialists are women, promotion delays may compound existing financial inequities, especially if institutional advancement criteria disproportionately reward professional patterns or achievements more accessible to male physicians. Understanding how promotion timing affects the NPV of earnings is critical to addressing inequities and guiding strategic decisions for both individuals and institutions.

The aim of this study was to analyze the NPV of lifetime earnings for academic general pediatricians and pediatric subspecialists. By applying standardized financial modeling to recent national compensation data, this study may offer evidence-based insights to inform physician career planning and guide institutional strategies that support equitable promotion and compensation in academic pediatric medicine.

## Methods

This cross-sectional study was conducted using aggregate-level analyses of an institutionally available, deidentified dataset. The study did not meet criteria for human participant research and did not require institutional review board approval or informed consent per the University of Arkansas for Medical Sciences. This study follows the Strengthening the Reporting of Observational Studies in Epidemiology (STROBE) reporting guideline.

### Data Source

We used 2024-2025 compensation data from the Association of Administrators in Academic Pediatrics (AAAP).^22^ The 25th, 50th, and 75th salary percentiles were collected across the ranks of assistant professor, associate professor, and full professor. A total of 29 pediatric subspecialties with complete data for all 3 ranks were included. The AAAP compensation dataset does not include physician demographic data.

### Career Length and Promotion Scenarios

We assumed full-time career lengths of 38 years for academic general pediatricians and those in emergency medicine, 37 years for sports medicine (1-year fellowship), and 35 years for other pediatric subspecialties (3-year fellowship). Estimates followed a usual educational timeline: high school graduation at age 18 years, 4 years each of college and medical school, 3 years of residency, optional fellowship, and retirement at age 67 years.

To reflect variability in physician careers, we also modeled 2 additional scenarios: (1) early retirement (career shortened by 10 years) and (2) part time (transition to 50% effort during the final 10 years of a standard career). We modeled 4 prototypical promotion trajectories: (1) early promotion (5 years as assistant professor, 5 years as associate professor, and remainder as full professor), (2) baseline promotion (7 years as assistant professor, 7 years as associate professor, and remainder as full professor), (3) stalled promotion (10 years as assistant professor, remainder as associate professor [no time as full professor]), and (4) no promotion (remained at assistant professor rank throughout entire career).

### Statistical Analysis

#### NPV Analysis

The NPV is a standard financial method for valuing investments over time by discounting future income to the present at a set rate. In general, and for this study, a positive NPV indicates profit, and a negative NPV indicates loss (in dollar amounts).^15^ For each specialty and promotion scenario, we projected annual salaries over the modeled career and calculated NPV by discounting future earnings to the present value. Year 1 salary equaled the current starting salary for the relevant rank and subspecialty. On the basis of a review of the past 10 years of salary data, we applied an average annual wage growth of 3% across ranks and percentiles. Upon promotion, salaries shifted to the current starting salary for the new rank, with 3% annual increases thereafter. The base case assumed 3% wage growth and a 4% annual discount rate, consistent with standard modeling. Sensitivity analyses varied wage growth from 0% to 6% to assess its effect on NPV. Thus, salary in year *t* was modeled as salary*_t_* = (starting salary for current rank) × (1 + 0.03)*^t^*^−1^, and the NPV of the lifetime earnings stream was


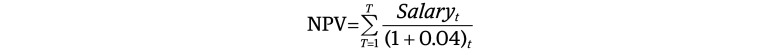
,

where *T* is the total career length in years (35, 37, or 38 years, depending on subspecialty). This approach incorporates annual raises and the declining present value of future earnings. The NPVs were calculated in constant 2025 dollars for each subspecialty and promotion scenario.

#### Sensitivity Analyses and Monte Carlo Simulation

We tested robustness with 1-way sensitivity analyses, varying wage growth from 0% to 5% (discount rate fixed at 4%) and discount rate from 1% to 5% (wage growth fixed at 3%). We modeled uncertainty for each promotion scenario using Monte Carlo simulations (10 000 iterations). Annual wage growth was drawn from a truncated normal distribution (mean, 3%; SD, 2%; range, 1%-5%) and the discount rate from a truncated normal distribution (mean, 4%; SD, 2%; range, 2%-6%). Randomly sampled parameters were applied to calculate NPVs, assessing variability and financial implications of promotion timing under plausible economic conditions. The NPV analyses were performed using Stata, version 19 (StataCorp LLC), and Monte Carlo simulations were performed using Python, version 3.11.5 (Python Software Foundation).

## Results

### Promotion Timing

**Figure 1.**
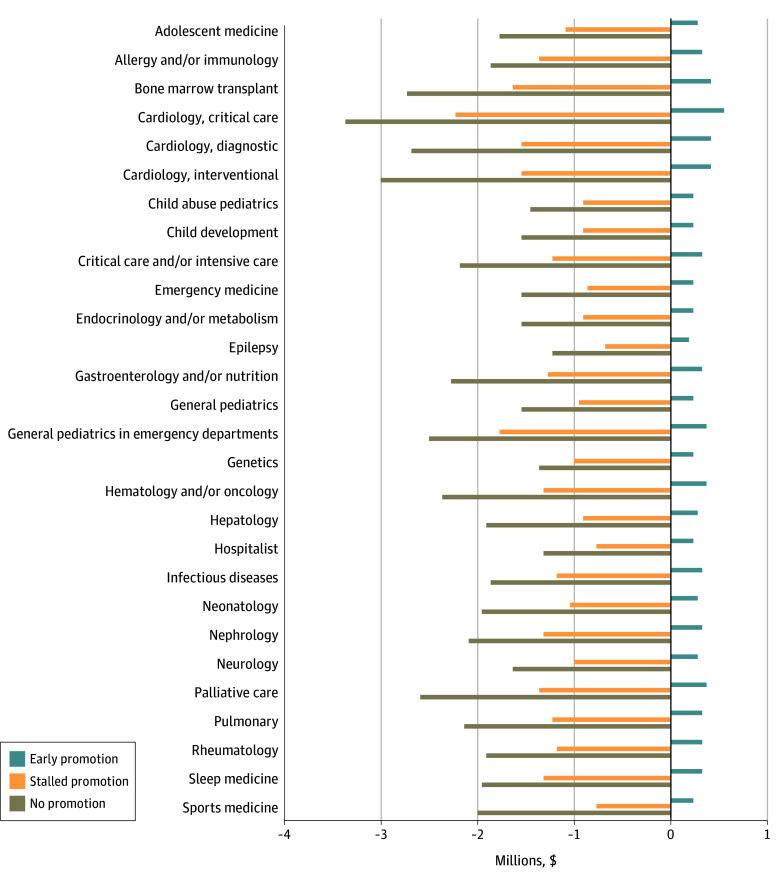
Difference in Net Present Value of Lifetime Earnings Early promotion (5 years as assistant professor, 5 years as associate professor, remainder as full professor), stalled promotion (10 years as assistant professor, remainder as associate professor, no time at full professor), and no promotion (remained as assistant professor throughout entire career) were compared with baseline promotion (7 years as assistant professor, 7 years as associate professor, remainder as full professor) for all pediatric specialties at the 50th salary percentile. Analysis was performed using a wage raise rate of 3% and discount rate of 4%.

A total of 19 111 academic pediatricians and pediatric subspecialists were included. Results of the NPV analysis for the early, stalled, and no promotion scenarios compared with the baseline promotion pathway are shown for the 50th salary percentile in Figure 1; results for the 25th and 75th percentiles are provided in eFigure 1 in Supplement 1. Across all academic pediatric specialties, early promotion increased the NPV of lifetime earnings compared with baseline promotion. In cardiac critical care, early promotion yielded a net gain of $554 719. In contrast, both stalled and no promotion reduced lifetime NPVs. In cardiac critical care, no promotion was associated with the largest loss of $3 351 314.

### Comprehensive Subspecialty Analysis

**Figure 2.**
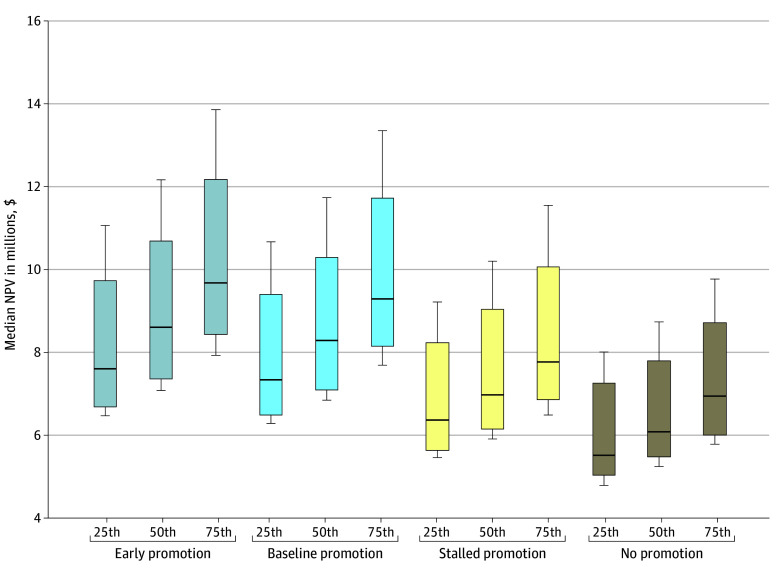
Median Lifetime Earning Potential for Early, Baseline, Stalled, and No Promotion at the 25th, 50th, and 75th Salary Percentiles Analyses were performed using a wage raise rate of 3% and discount rate of 4%. The horizontal bar inside the boxes indicates the median, and the lower and upper ends of the boxes are the first and third quartiles, respectively. The whiskers indicate values within the minimum and maximum range. NPV indicates net present value.

Early promotion consistently produced the highest NPV, regardless of salary percentile (median NPV, $7 621 223.50 for early promotion vs $5 541 729.90 for no promotion at the 25th percentile) (Figure 2). Notably, pediatricians who experienced stalled or no promotion (median NPV, $7 789 262.07 and $6 967 428.72, respectively), despite earning at the 75th percentile, often had lower NPVs than those promoted early (median NPV, $9 694 178.89) (Figure 2). Although NPV increased stepwise with higher salary percentiles, the influence of promotion timing was more pronounced. For example, the NPV for baseline promotion at the 25th percentile exceeded that of no promotion at the 75th percentile. Stalled promotion reduced the median NPV by $500 000 to $1.5 million; no promotion led to losses exceeding $2 million to $3 million in high-paying subspecialties, such as cardiology, critical care, and neonatology.

### Monte Carlo Simulation

Monte Carlo simulations revealed distinct distributions of NPV by promotion trajectory (eFigure 2 in Supplement 1). Random variation in wage growth and discount rate did not meaningfully alter the hierarchy of outcomes. No-promotion scenarios yielded the lowest NPV, clustering at a mean (SD) of $7 093 248.02 ($475 237.71). Stalled promotion produced slightly higher values, centered at a mean (SD) of $8 009 890.81 ($554 284.58), while baseline promotion peaked at a mean (SD) of $9 364 986.25 ($695 347.50). Early promotion showed the highest NPV, with values concentrated at a mean (SD) of $9 715 439.95 ($696 610.71). These findings confirm a robust, positive association between earlier promotion and higher lifetime earnings.

### Wage Growth Sensitivity Analyses

**Figure 3.**
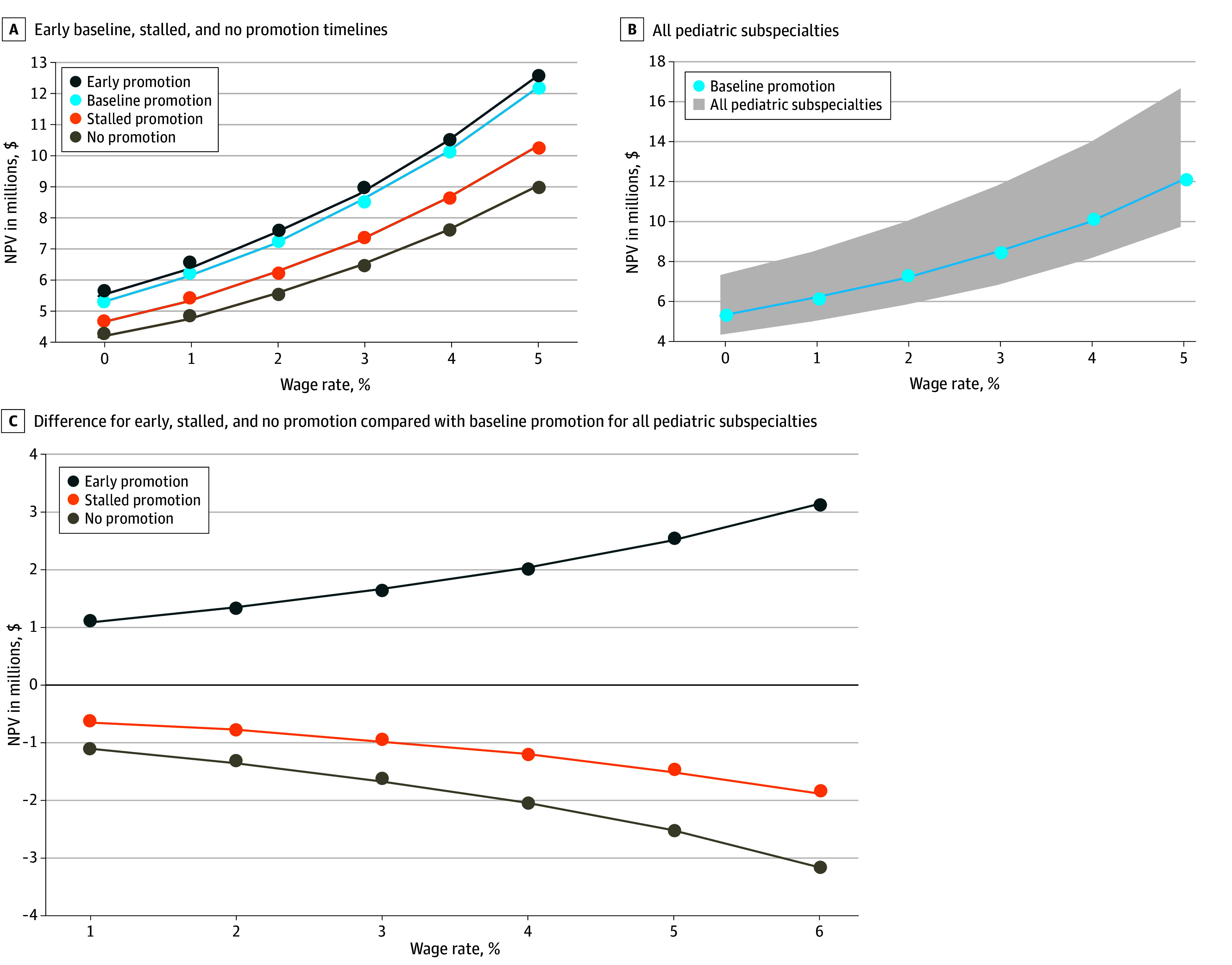
Mean Net Present Value (NPV) at the 50th Salary Percentile for All Pediatric Subspecialities Compared With Wage Rate Across All Promotion Timelines Analyses were performed using a wage raise rate of 3% and discount rate of 4%.

Early promotion was associated with the highest NPV at each wage growth rate, reaching $12 420 543 million at a 5% growth rate compared with $8 940 183 million for no promotion (Figure 3A). A stepwise pattern was evident between each promotion category. Figure 3B displays the NPV trajectory for baseline promotion at the 50th salary percentile, with the shaded region depicting the range of NPVs across all pediatric subspecialties, each showing a positive correlation between wage growth and NPV. Figure 3C shows mean NPV differences compared with the baseline promotion timeline. Early promotion yielded the largest gains, while stalled and no promotion were consistently associated with losses. Figure 3C also shows that small changes in wage growth have an outcome worth thousands, such as wage growth from 4% to 5% for early promotion yielding an increase of $34 416.

### Career Length

**Figure 4.**
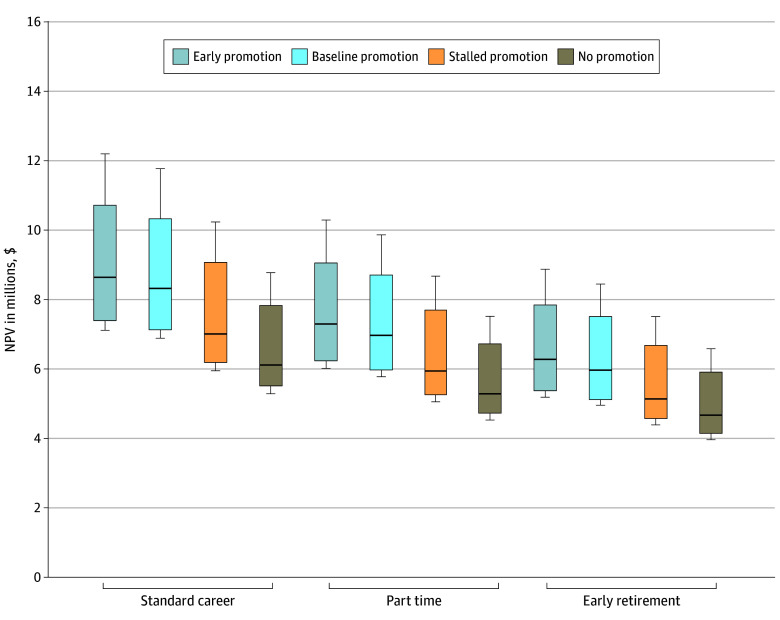
Net Present Value (NPV) of Lifetime Earnings Based on Career Length and Promotion Timelines The standard career length was defined as 38 years for academic general pediatrics and pediatricians working in emergency departments, 37 years for sports medicine, and 35 years for all other subspecialties. Part-time career length was defined as physicians transitioning to 50% time 10 years before retirement. Early retirement career length was defined as physicians ending their careers 10 years earlier than the standard career length. Analyses were performed using a wage raise rate of 3% and discount rate of 4%. The horizontal bar inside the boxes indicates the median, and the lower and upper ends of the boxes are the first and third quartiles, respectively. The whiskers indicate values within the minimum and maximum range.

For standard career length, early promotion yielded the highest median NPV ($8.6 million), followed by baseline ($8.3 million), stalled ($7.0 million), and no ($6.1 million) promotion (Figure 4). A similar ranking was observed in the part-time model. For early retirement, the median NPVs decreased across all categories but maintained the same pattern (early promotion, $6.3 million; baseline, $5.9 million; stalled, $5.1 million; no promotion, $4.7 million).

## Discussion

This cross-sectional study evaluated the association of academic promotion scenarios with the NPV of lifetime earnings for academic general pediatricians and pediatric subspecialists and produced 4 key findings. First, promotion timing substantially influenced lifetime earnings, regardless of subspecialty or salary percentile. Second, physicians who experienced delayed or no promotion incurred substantial financial losses, often amounting to millions of dollars, over the course of their careers. Third, variation in NPV across pediatric subspecialties was consistently shaped by promotion status, often more than by salary percentile alone. Fourth, physicians who were never promoted would need to work approximately 10 additional years to achieve the same lifetime NPV as peers who followed a baseline promotion trajectory. These findings carry important implications for addressing long-standing disparities in physician compensation, particularly those rooted in specialty, gender, and institutional structures, and for safeguarding the future of the pediatric workforce.

While prior studies have examined the financial implications of pediatric subspecialty training, most assumed uniform promotion trajectories.^3,11,12,17^ Catenaccio et al^14^ recently modeled the association of early-career promotion delays, and our study expands on this by analyzing full-career promotion trajectories across subspecialties, salary percentiles, and career lengths. In a separate analysis, Catenaccio et al^11^ showed that the inpatient-oriented and procedure-oriented subspecialties, eg, cardiology and critical care, yield higher lifetime earnings than outpatient-focused fields, eg, adolescent medicine, with the financial value of fellowship training ranging from a $1.6 million loss to an $850 000 gain, depending on subspecialty. Moreover, this disparity has widened over time, from $1.4 million in 2007 to 2008 to more than $2.3 million in 2018 to 2019.^23^ These models, however, assumed standard promotion timelines. We extend this work by incorporating variable promotion trajectories and showing that promotion timing itself may be a powerful and independent driver of long-term financial well-being. For example, among pediatric cardiac critical care physicians, a no-promotion scenario at the 50th salary percentile was associated with a $3.3 million reduction in lifetime NPV compared with baseline promotion, despite the subspecialty’s relatively high compensation. In some cases, the influence of promotion timing outweighed that of subspecialty choice.

Our results also reveal that promotion status may influence earnings more than salary percentile. Across subspecialties, pediatricians promoted early and paid at the 25th percentile outearned those remaining at assistant professor at the 75th percentile. For instance, the median NPV for early promotion at the 25th percentile was $7.6 million, exceeding the $6.9 million NPV of physicians who were never promoted but earned at the 75th percentile. These results underscore that promotion trajectory, more than salary level alone, substantially influences financial outcomes in academic pediatrics. The implications are magnified when considering flexibility in work-life balance: A physician promoted early could retire 10 years earlier vs being never promoted and still accumulate greater lifetime earnings. These findings highlight the importance of institutional policies that support timely promotion to enhance retention, morale, and financial well-being among pediatric faculty.

Our findings also carry substantial implications for gender equity. Although women comprise 64% of the pediatric workforce, only 40% of full professors and less than 20% of department chairs are women.^24^ Women in academic medicine are consistently less likely to be promoted than men, even after adjusting for experience, productivity, and specialty.^20,25,26^ Importantly, women are also promoted more slowly. One national analysis found that among faculty promoted to associate professor, women experienced a median delay of 214 days compared with men, even after adjusting for key academic factors.^27^ Our findings show that delays in promotion have a substantial negative financial outcome, often reducing lifetime earnings by more than $1 million to $3 million. Taken together, these data suggest that unequal promotion timelines may be a critical and underrecognized driver of the persistent gender pay gap in academic medicine.^28,29^

These disparities are compounded by the tendency of lower-paying pediatric subspecialties having higher female representation. Catenaccio et al^28^ found that for every 1% increase in the proportion of women in a pediatric subspecialty, the average lifetime earnings decreased by approximately $55 000, suggesting a systemic undervaluation of female-dominant fields. Institutional reforms that systematically support women’s progression through academic ranks, including mentorship, transparency in criteria, and equitable access to leadership roles, are crucial to narrowing these gaps. Our findings offer a quantitative framework that may help institutions recognize how delays in promotion disproportionately harm women and reinforce structural inequities in academic pediatrics.

Our analysis adds to the broader discussion of compensation differences between academic pediatric and adult subspecialists. Pediatric subspecialists earn approximately 25% less than their adult subspecialist counterparts, resulting in a median lifetime NPV shortfall exceeding $1.2 million.^23^ This disparity stems from several factors, including greater reliance on Medicaid reimbursement, lower procedural volumes, and differences in case complexity.^4^ While addressing systemic factors would require policy-level interventions, timely promotion is a modifiable institutional factor that may partially mitigate earning gaps. Early promotion may also offer physicians the flexibility to pursue part-time work or early retirement without incurring major financial penalties, especially for those balancing caregiving responsibilities or seeking improved work-life integration. In this way, promotion may serve as both a financial buffer and a tool to mitigate broader structural inequities.

While often viewed as a marker of scholarly achievement or leadership potential, our findings suggest that promotion also represents a pivotal financial inflection point. Institutional support, through mentorship, transparent criteria, and protected academic time, varies widely across programs, and the downstream impact of this variability has not been systematically studied.^30,31^ Promotion outcomes can be influenced by implicit bias, departmental culture, resource allocation, and mentorship infrastructure.^32^ Our results suggest that unequal promotion support may have profound downstream financial implications. Future qualitative studies should explore promotion barriers, especially among women, underrepresented minority groups, and physicians in lower compensated specialties. Additionally, promotion delays may contribute to dissatisfaction, burnout, and attrition, challenges that already disproportionately burden pediatric departments struggling with recruitment and retention.

While our model used a 7-plus-7–year promotion timeline (assistant to associate professor, then associate to full professor) as the baseline, this trajectory may not reflect usual academic experiences. Association of American Medical Colleges data indicate that only 34% of physician faculty are promoted to associate professor and 50% to full professor within 10 years, suggesting that delayed promotion is common.^33^ However, a national pediatrics cohort study reported median promotion times of 5.8 years to associate professor and 9.4 years to full professor.^34^ These conflicting data highlight the uncertainty and variability in actual promotion patterns across institutions and specialties. Further research is needed, especially given our findings that delays in promotion may reduce lifetime earnings by $1 million to $3 million. Institutional differences in promotion timelines may have major financial implications, making promotion policy and culture an underrecognized driver of long-term compensation.

Future research should focus on collecting actual institutional data on promotion rates and timelines across pediatric subspecialties. Understanding how institutional culture and policies shape advancement would be critical for developing strategies to promote equity and long-term sustainability. Incorporating promotion-adjusted models into future comparisons between pediatric and adult specialties could also present a more accurate picture of structural disparities. Promotion policies should be seen not only as milestones in individual careers but also as strategic levers for supporting the long-term viability of the pediatric academic workforce.

### Limitations

This study had some limitations. Our analysis relied on assumptions about career length, retirement age, salary growth, and promotion timelines. Physicians may pursue additional fellowship years or retire earlier, which may influence lifetime NPV through total career duration. To account for this possibility, we modeled early retirement and part-time scenarios and found that the financial outcomes of promotion remained robust. Our assumed 3% annual salary growth was based on recent historical AAAP data. Although absolute NPVs varied across growth rates, the relative influence of promotion timing persisted. We also assumed that salary increases with promotion maintain the same percentile within the AAAP dataset. It is unclear whether actual salary increases at promotion occur as discrete jumps or more gradual changes influenced by merit, retention efforts, or institutional variation. Although introducing some imprecision into our model, it did not alter our central conclusion that promotion timing is associated with shaping financial outcomes in academic pediatrics. We were also unable to model physicians who pursued part-time roles earlier in their careers.

## Conclusions

This cross-sectional study found that promotion timing is associated with the NPV of lifetime earnings for academic general pediatricians and pediatric subspecialists. Even after accounting for salary percentile, career length, and subspecialty, promotion trajectory remained one of the dominant drivers of long-term financial outcomes. These findings underscore that academic promotion is not merely a symbolic milestone but a critical factor in shaping financial security, equity, and workforce sustainability. To strengthen the pediatric academic workforce, institutions must invest in transparent promotion pathways, meaningful mentorship, and equitable advancement policies that support faculty throughout their careers.

## Notes

Supplement 1. **eFigure 1.** Differences in Net Present Value of Lifetime Earnings **eFigure 2.** Monte Carlo Simulation of Lifetime Earnings Based on Early, Baseline, Stalled, and No Promotion Timelines

Supplement 2.
Data Sharing Statement
