# Supplementary material for: Academic General and Subspecialty Pediatric Promotion Timelines and Lifetime Earnings
Source: JAMA Netw Open. 2025 Oct 31;8(10):e2540875. doi: 10.1001/jamanetworkopen.2025.40875 (PMC12579352; doi:10.1001/jamanetworkopen.2025.40875)
Supplement: Supplement 1. — eFigure 1. Differences in Net Present Value of Lifetime Earnings eFigure 2. Monte Carlo Simulation of Lifetime Earnings Based on Early, Baseline, Stalled, and No Promotion Timelines [file jamanetwopen-e2540875-s001.pdf]

Supplemental Online Content

Ehsan L, Almasri M, Joshi, K, Celik B, Hong Y, Daily J. Academic general and subspecialty pediatric promotion timelines and lifetime earnings. *JAMA Netw Open*. 2025;8(10):e2540875. doi:10.1001/jamanetworkopen.2025.40875

eFigure 1. Differences in Net Present Value of Lifetime Earnings
eFigure 2. Monte Carlo Simulation of Lifetime Earnings Based on Early, Baseline, Stalled, and No Promotion Timelines

This supplemental material has been provided by the authors to give readers additional information about their work.

© 2025 Ehsan L et al. *JAMA Network Open*.

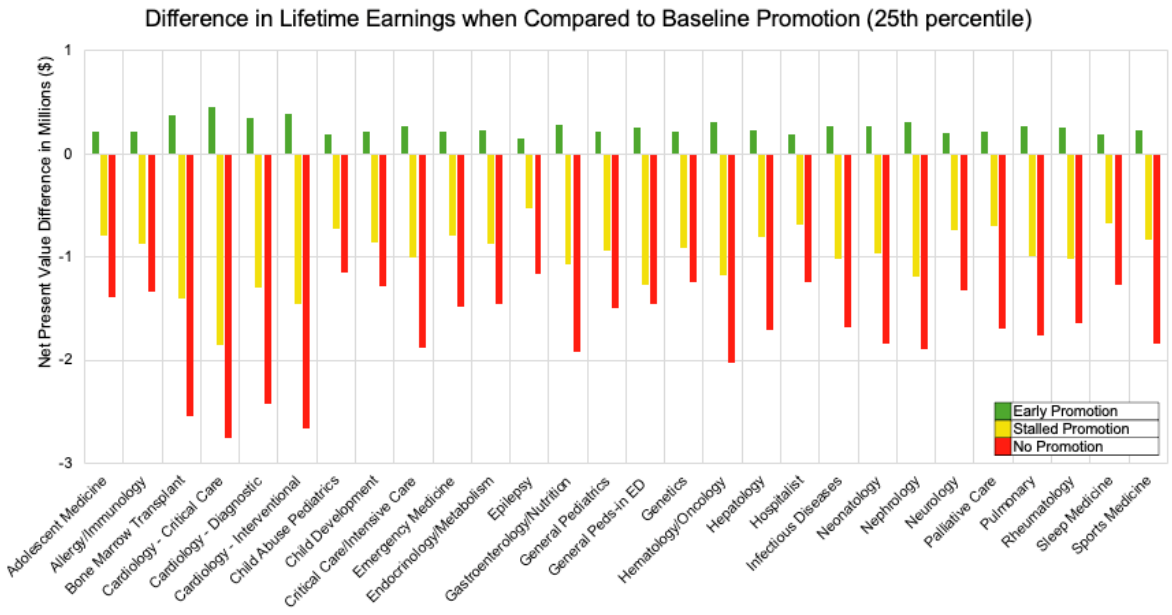

eFigure 1. Difference in Net Present Value of Lifetime Earnings

A: Difference in net present value of lifetime earnings for early (5 years as Assistant Professor, 5 years as Associate Professor, remainder as Full Professor), stalled (10 years as Assistant, remainder as Associate Professor, no time at Full Professor rank), and no promotion (remain at Assistant Professor rank throughout entire career) compared to baseline promotion (7 years as Assistant, 7 years as Associate, remainder as Full Professor) for all pediatric specialties at 25th salary percentile. Analysis was performed using a wage raise rate of 3% & discount rate of 4%. Key–General Peds-in ED: academic pediatricians working in emergency departments.

© 2025 Ehsan L et al. *JAMA Network Open*.

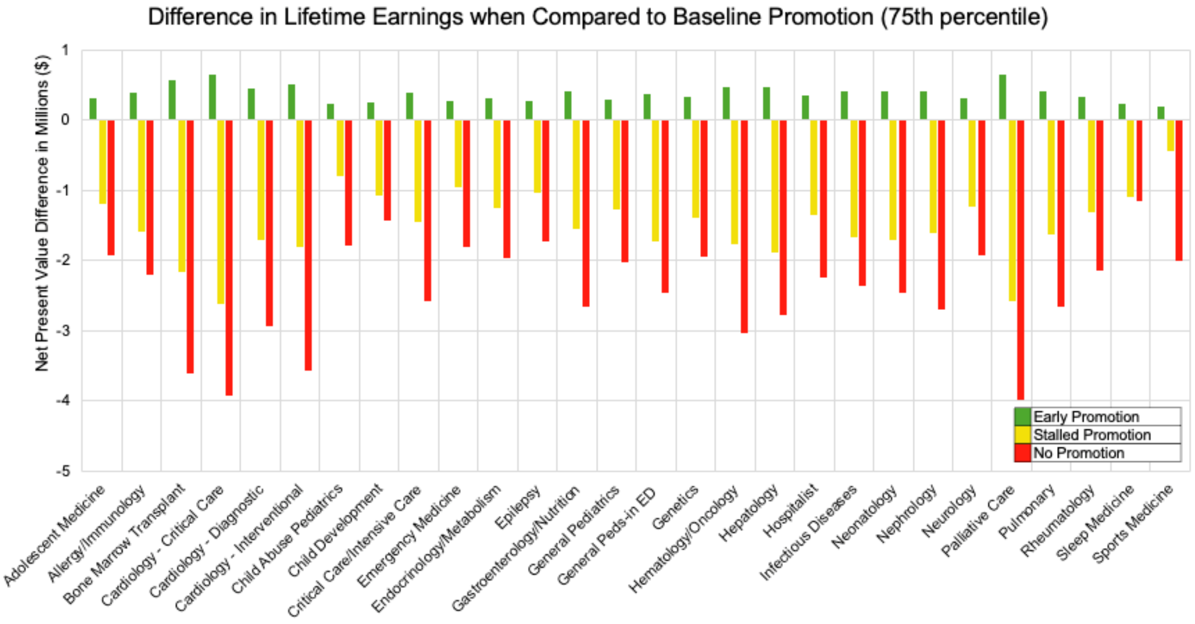

B: Difference in net present value of lifetime earnings for early (5 years as Assistant Professor, 5 years as Associate Professor, remainder as Full Professor), stalled (10 years as Assistant, remainder as Associate Professor, no time at Full Professor rank), and no promotion (remain at Assistant Professor rank throughout entire career) compared to baseline promotion (7 years as Assistant, 7 years as Associate, remainder as Full Professor) for all pediatric specialties at 75th salary percentile. Analysis was performed using a wage raise rate of 3% & discount rate of 4%. Key–General Peds-in ED: academic pediatricians working in emergency departments.

© 2025 Ehsan L et al. *JAMA Network Open*.

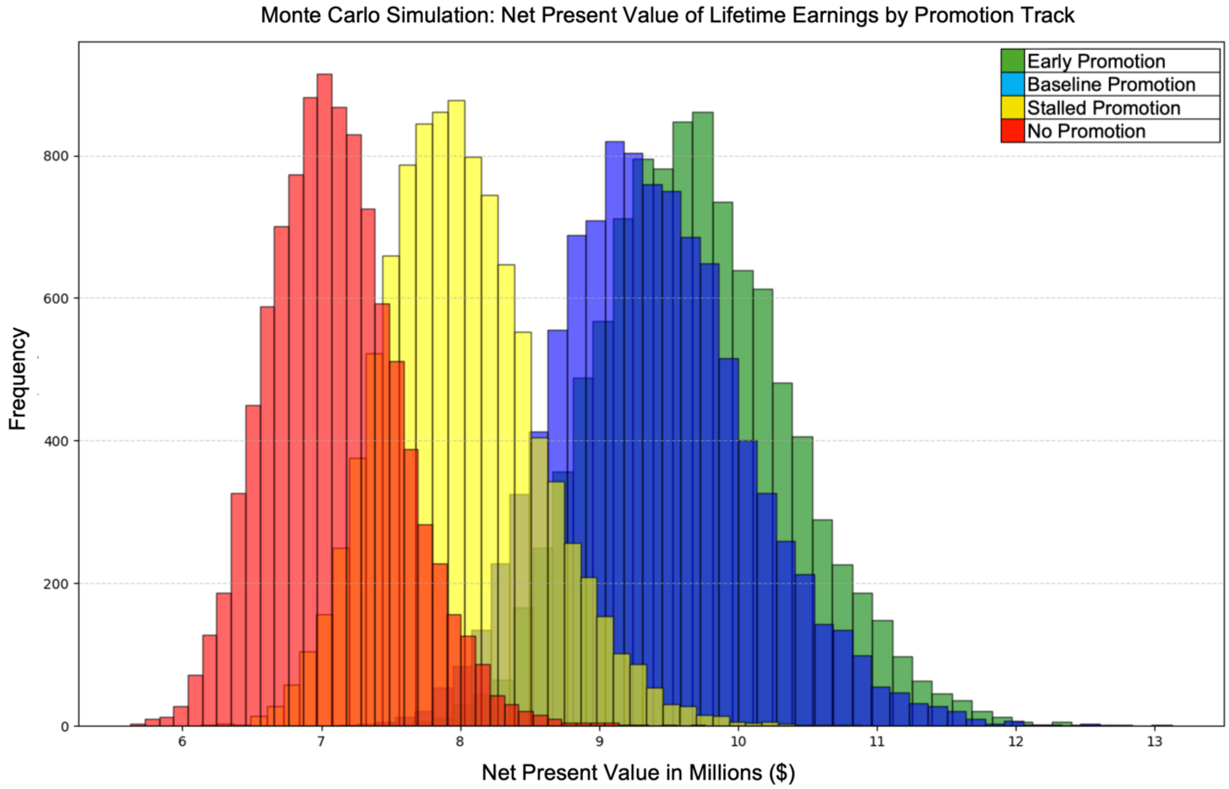

eFigure 2. Monte Carlo Simulation of Lifetime Earnings Based on Early, Baseline, Stalled, and No Promotion Timelines.

Analysis was done for raise 3%, discount 4%.

© 2025 Ehsan L et al. *JAMA Network Open*.
