# Supplementary material for: Academic General and Subspecialty Pediatric Promotion Timelines and Lifetime Earnings
Source: JAMA Netw Open. 2025 Oct 31;8(10):e2540875. doi: 10.1001/jamanetworkopen.2025.40875 (PMC12579352; doi:10.1001/jamanetworkopen.2025.40875)
Supplement: Supplement 2. — Data Sharing Statement [file jamanetwopen-e2540875-s002.pdf]

Data Sharing Statement

Data
Data available: Yes
Data types: Data (not involving human participants)
How to access data: Aggregated analyses of the data will be available upon request from the corresponding author.
When available: With publication

Supporting Documents
Document types: None

Additional Information
Who can access the data: Anyone requesting the data
Types of analyses: For any purpose
Mechanisms of data availability: Without investigator support
